# Magnetite/MXene (Fe_3_O_4_/Ti_3_C_2_) Nanocomposite as a Novel Adsorbent for Environmental Remediation of Malachite Green Dye

**DOI:** 10.3390/molecules29061372

**Published:** 2024-03-19

**Authors:** Amal M. Alkhudaydi, Ekram Y. Danish, Mohamed Abdel Salam

**Affiliations:** 1 Department of Chemistry, Faculty of Science, King Abdulaziz University, P.O. Box 80200, Jeddah 21589, Saudi Arabia; mool-khu11@hotmail.com (A.M.A.); eydanish@kau.edu.sa (E.Y.D.); 2 Department of Chemistry, Faculty of Science, Taif University, P.O. Box 11099, Taif 21944, Saudi Arabia

**Keywords:** MXene, Fe_3_O_4_/Ti_3_C_2_ nanocomposite, removal, malachite green dye, remediation

## Abstract

In this work, a novel adsorbent called magnetite/MXene (Fe_3_O_4_/Ti_3_C_2_) nanocomposite was prepared, characterized, and applied for the removal of organic dye, malachite green dye (MG), from both real water and model solutions. Numerous techniques were used to characterize the prepared Fe_3_O_4_/Ti_3_C_2_ nanocomposite: XRD, SEM, TEM, FTIR, and surface area analysis. The outcomes showed that the Al layer had been selectively etched, that the MAX phase (Ti_3_AlC_2_) had been transformed into layered Ti_3_C_2_ MXene, that the cubic Fe_3_O_4_ phase had been prepared, and that the prepared Fe_3_O_4_ NPs had been evenly distributed on the MXene surface. Also, SEM pictures showed the successful etching of the MAX phase and the formation of the ultrathin multi-layered MXene, which the Fe_3_O_4_ NPs covered upon forming the Fe_3_O_4_/Ti_3_C_2_ nanocomposite at the surface and inside the ultrathin multi-layered MXene. The effect of different operational parameters affecting the removal process was explored and optimized. The MG dye was removed mostly within 60 min, with a 4.68 mg/g removal capacity using 5 mg of the Fe_3_O_4_/Ti_3_C_2_ nanocomposite. The removal was examined from both kinetic and thermodynamic perspectives, and the findings demonstrated the spontaneity of the removal process as well as the applicability of fractal-like pseudo-first-order and fractal-like pseudo-second-order kinetics when compared to other kinetics models. The Fe_3_O_4_/Ti_3_C_2_ nanocomposite was used to remove MG dye from real spiked environmental water samples, and the results revealed the successful remediation of the real samples from the organic dye by the Fe_3_O_4_/Ti_3_C_2_ nanocomposite. Accordingly, Fe_3_O_4_/Ti_3_C_2_ nanocomposite could be considered a potential adsorbent for the environmental remediation of polluted water.

## 1. Introduction

Most consumer items, including textiles, paper, leather, food, and cosmetics, are colored using synthetic organic dyes that, when combined with other auxiliary chemicals, form large discharges that threaten aquatic life and humans with poisonous, carcinogenic, and mutagenic effects, in addition to their great susceptibility to chelate metal ions, increasing their toxicity to fish and other creatures [1]. Additionally, synthetic organic dyes in water bodies absorb light and prevent light penetration, decreasing photosynthetic activity and preventing the growth of aquatic vegetation [2].

The removal of organic dyes can be accomplished by utilizing a variety of treatment methods, including ion exchange, precipitation, coagulation–flocculation, filtration, liquid–liquid extraction, electrochemical methods, and adsorption [3], especially with carbon-based materials for adsorption and separation [4,5]. Adsorption is considered one of the most efficient removal techniques as it has numerous advantages, including a simple design, reusability of adsorbents/adsorbate, low cost, ease of operation, fewer chemical requirements, high efficiency, and a short time of removal [6].

One of the great challenges facing the application of the adsorption process is the search for new adsorbents with high efficacy. Therefore, exploring novel adsorbents is still a prodigious task for the scientific community. Nothing excites the community of material scientists more than the discovery of a new family of two-dimensional (2D) nanomaterials, such as transition metal dichalcogenides (e.g., MoS_2_ and WS_2_), black phosphorous layered double hydroxides (LDHs), g-C_3_N_4_, boron nitride, graphene, graphene oxide, metal–organic frameworks (MOFs), and MXenes, which are characterized by a wide range of potential compositions and tailorable features for varied uses [7], especially in applied environmental cleaning [8]. Moreover, the layered 2D materials can easily attach to the nano-sized encapsulated particles through strict layer spaces for various applications [9].

MXene is the most recent member of the 2D nanomaterials family; due to their strong negative surface charge, high chemical stability, adjustable chemistry, and high hydrophilicity, MXenes can be employed in a wide range of applications, such as absorbents, energy storage, supercapacitors, catalysis, gas sensors, polymer-based composites, electromagnetic interference (EMI) shielding antibiosis, and lubrication [10]. Many surface modification techniques have been cleverly developed recently to increase the potential of MXenes in various sectors, including surface-initiated polymerization and single-heteroatom doping [11]. MXenes were recently applied in dye removal [12,13]; for instance, they were used for eliminating methylene blue dye from water [14] and were also used for the removal of malachite green dyes [15]. MXene nanosheets were directly deposited on commercially available filters for fast and efficient dye removal, and the results indicated outstanding performance [16]. This demonstrates the possibility of using MXenes in the adsorption and environmental remediation of dyes from water. Recently, MXene modification was explored to enhance extraordinary properties [17,18,19]. Most recently, MXene/carbon foam hybrid aerogel (MCF) was synthesized and used for methylene blue (MB) and Congo red (CR) [20], MXene/NiFeMn-layered double hydroxide decorated gelatin for the removal of Congo red [21], and porous heterostructured MXene/biomass-activated carbon composites were synthesized and used for the removal of three anionic azo dyes: allure red, Congo red, and sunset yellow [22]. In the recent past, the field of environmental remediation has paid considerable attention to magnetite nanoparticles (Fe_3_O_4_ NPs) due to their tendency to aggregate and form clusters [23]. Magnetite nanoparticles (Fe_3_O_4_) are among the most promising transition metal oxides that are used for the modification of various adsorbents [24,25,26] because of their advantages of having excellent theoretical unique capacity, affordability, and nontoxicity.

The novelty of the current research is to develop a unique, efficient, and reliable solid hybrid adsorbent based on magnetite and MXene for the environmental remediation of polluted water with organic dyes such as malachite green dye.

Accordingly, in this study, magnetite nanoparticles were used for the modification of MXene to form the magnetite/MXene (Fe_3_O_4_/Ti_3_C_2_) nanocomposite and used to remove malachite green (MG) dye, as an example of synthetic organic dye, from a simulated dye aqueous solution. The adsorption process was investigated based on the operational factors adsorbent mass, interaction time, temperature, solution pH, and ionic strength, and the mechanism was explored using several adsorption kinetics models, in addition to thermodynamics.

## 2. Results and Discussion

### 2.1. Characterization 

**Figure 1:**
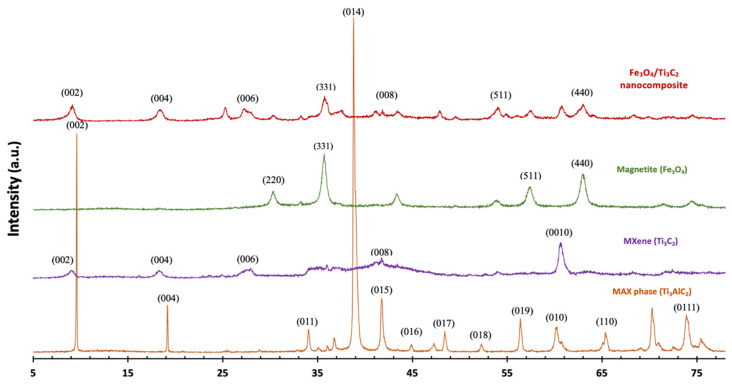
XRD patterns of MAX phase (Ti_3_AlC_2_), MXene (Ti_3_C_2_), magnetite (Fe_3_O_4_), and Fe_3_O_4_/Ti_3_C_2_ nanocomposite.

The XRD patterns of the Ti_3_AlC_2_ MAX, exfoliated Ti_3_C_2_ MXene, Fe_3_O_4_ nanoparticles, and Fe_3_O_4/_Ti_3_C_2_ nanocomposite are shown in Figure 1. At 2θ = 39°, the peak attributed to Ti_3_AlC_2_′s (1 0 4) peak disappeared after the procedure of HF exfoliation. Additionally, the peak at (0 0 2) for 2D Ti_3_C_2_ changed from 2θ = 9.7° to 2θ = 9.1°, indicating that the Ti_3_AlC_2_ phase transformed into layered Ti_3_C_2_ MXene and that the Al layer was carefully etched. There are four diffraction peaks for the generated Fe_3_O_4_, at 30.2°, 35.5°, 57.2°, and 62.8°, which belong to the cubic Fe_3_O_4_ phase’s (2 2 0), (3 1 1), (5 1 1), and (4 4 0) planes, respectively (JCPDS card no. 19-0629). By applying the Scherrer Formula and utilizing the (3 1 1) peak’s half-peak width, it is expected that the produced Fe_3_O_4_ crystallite size is about 27.5 nm. There are no discernible XRD peaks in response to new crystalline phases in the Fe_3_O_4_/Ti_3_C_2_ nanocomposite; instead, the XRD patterns are composed solely of cubic Fe_3_O_4_ and pure Ti_3_C_2_. This means that the nanocomposite is a true combination of Ti_3_C_2_ MXene and Fe_3_O_4_ nanoparticles, which is in line with previously published work [27].

**Figure 2:**
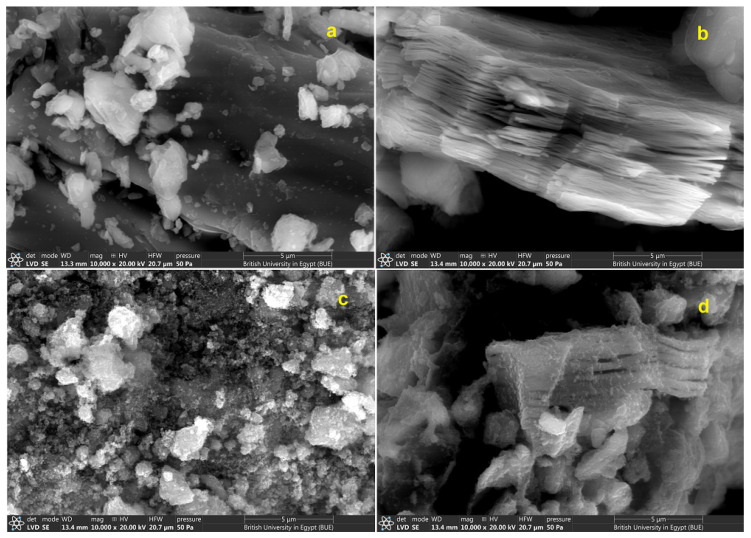
SEM micrographs of (**a**) MAX phase, (**b**) MXene, (**c**) magnetite, and (**d**) Fe_3_O_4_/Ti_3_C_2_ nanocomposite.

The MAX phase, prepared Ti_3_C_2_ MXene, prepared Fe_3_O_4_ NPs, and Fe_3_O_4_/Ti_3_C_2_ nanocomposite microstructures were examined using scanning electron microscopy, and the micrographs are presented in Figure 2. The images of the MAX phase (Ti_3_AlC_2_) before (Figure 2a) and following an HF etching procedure that eliminated the Al atoms to produce the exfoliated 2D MXene nanosheets (Ti_3_C_2_) (Figure 2b) possessing a few nanometers’ thickness are shown. The MAX phase was characterized by an uneven surface and a closely packed structure, whereas the resulting exfoliated 2D MXene nanosheets were ultrathin and multi-layered, characterized by clear sharp edges, confirming the success of the etching process because of the weak Ti–Al bonds compared to the Ti–C bonds within the MAX phase. Also, due to the extreme reactivity of the Al atoms, they could be easily etched and removed from the Ti_3_AlC_2_ structure and form the 2D MXene nanosheets (Ti_3_C_2_). Moreover, the ultrathin sheets are parallel to one another, which suggests that the basal planes and Ti_3_AlC_2_ etching directions are parallel, which is similar to previous studies [28,29,30]. Figure 2c shows the Fe_3_O_4_ NPs, which are characterized by a large quantity of agglomerated nanoparticles with a very small size. Figure 2d displays the uniform dispersion of Fe_3_O_4_ nanoparticles both on the exterior and within the incredibly thin multi-layered MXene. The Fe_3_O_4_ nanoparticles could be easily inserted between the layers of Ti_3_C_2_ MXene or adsorb on their surface.

**Figure 3:**
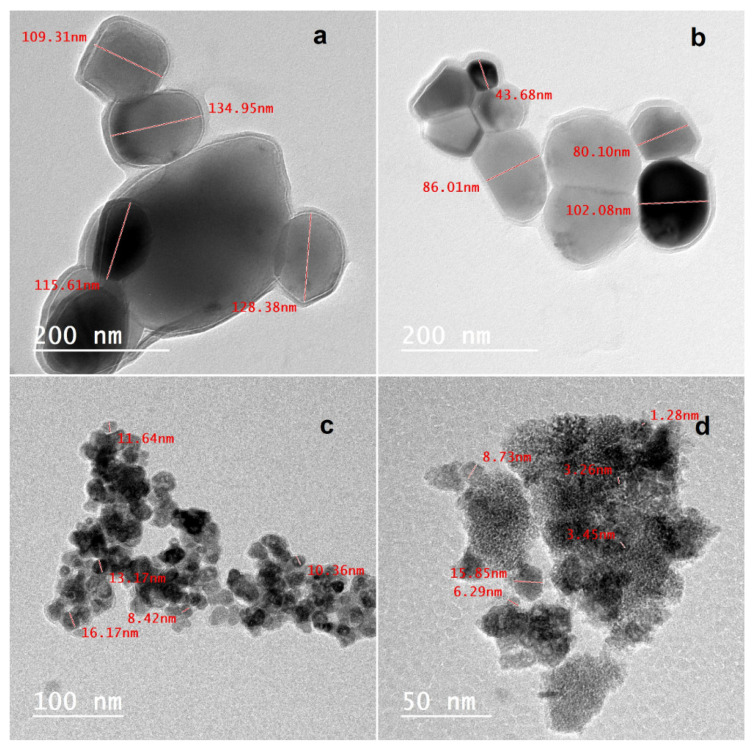
TEM micrographs of (**a**) MAX phase, (**b**) MXene, (**c**) magnetite, and (**d**) Fe_3_O_4_/Ti_3_C_2_ nanocomposite.

**Figure 4:**
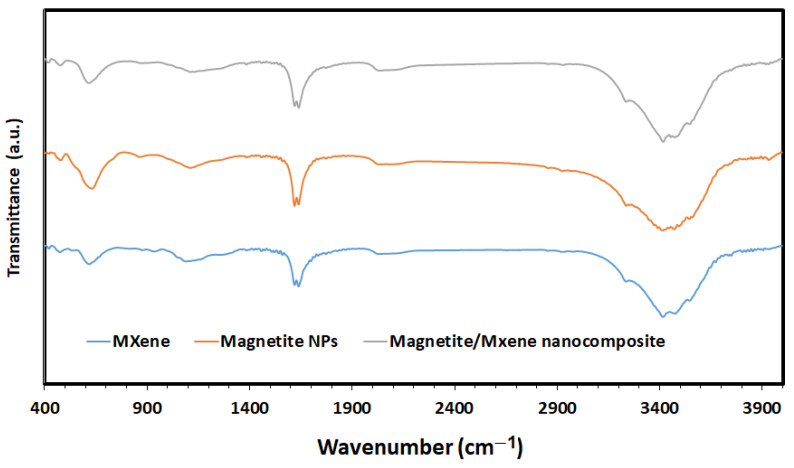
FTIR spectra of MXene, magnetite NPs, and Fe_3_O_4_/Ti_3_C_2_ nanocomposite.

Further examination of the MAX phase, Ti_3_C_2_ MXene, Fe_3_O_4_ NPs, and Fe_3_O_4_/MXene nanocomposite was performed using TEM. Figure 3a,b show the MAX phase and Ti_3_C_2_ MXene nanosheets, which demonstrated overlapped and nearly transparent sheets with a few nanometers’ diameter. It is evident that the diameter of the MXene is smaller than the MAX phase due to the etching process, which decreased the size of MXene compared to the MAX phase: 78 nm and 122 nm, respectively. Additionally, the aggregation of Fe_3_O_4_ NPs with an average size of particles of 11 nm was visible in the TEM image, and the homogeneous distribution of the Fe_3_O_4_ NPs over the MXene nanosheets is displayed in Figure 3d. FT-IR analyses of the MAX phase, Ti_3_C_2_ MXene, Fe_3_O_4_ NPs, and Fe_3_O_4_/MXene nanocomposite were explored to provide details on the produced nanomaterials’ composition and surface functionality. The FTIR spectrum of the Ti_3_AlC_2_ MAX phase is displayed in Figure 4, with pronounced bands at 474, 617, 1087, and 1541 cm^−1^ corresponding to the TiO_2_, Ti–O, Al–O, and CH groups, respectively, in addition to the strong vibration band of the –OH group at 3500 cm^−1^ due to the adsorbed external water. In the case of MXene, the vibrational stretching of the highly hydrogen-bonded –OH group and the bending vibration of the C–OH bond, respectively, could be the cause of the absorption peaks at 1638 and 3686 cm^−1^ because MXene’s hydrophilic nature causes it to absorb external water molecules or strongly coordinated H_2_O. Such distinct –OH groups’ stretching vibrational patterns may also be associated with Ti^4+^ cations. Moreover, Ti–O–Ti linkage and Ti–O bonding deformation vibrations account for the maxima at 877 and 617 cm^−1^, respectively. Furthermore, the band at 617 cm^−1^ might be a representation of rocking or twisting Ti–C out-of-plane vibrations [31,32]. The FT-IR spectrum of Fe_3_O_4_ nanoparticles showed absorption bands at 550–1650 cm^−1^ due to Fe–O group flexural vibrations and strong vibration bands of the –OH group at 1638 and 3500 cm^−1^ due to adsorbed external water. The FTIR spectrum of the Fe_3_O_4_/MXene nanocomposite showed most of the characteristic absorption bands of both MXene and the Fe_3_O_4_ NPs. The specific surface area values of the samples were calculated from the nitrogen gas adsorption/desorption isotherms at 77 K, and the values were 10.40, 40.25, 59.89, and 32.86 m^2^/g for the MAX phase, Ti_3_C_2_ MXene, Fe_3_O_4_ NPs, and Fe_3_O_4_/MXene nanocomposite, respectively. There was a significant increase in the MAX phase’s specific surface area following HF etching and the formation of exfoliated 2D nanosheets of MXene, from 10.40 to 40.25 m^2^/g. Upon the modification of MXene with the Fe_3_O_4_ NPs and the formation of the Fe_3_O_4_/MXene nanocomposite, this BET surface area was slightly reduced from 40.25 to 32.86 m^2^/g due to the distribution of the Fe_3_O_4_ NPs over the MXene nanosheets, as was presented earlier by the SEM (Figure 2) and TEM (Figure 3) analyses.

### 2.2. Adsorption Studies

**Figure 5:**
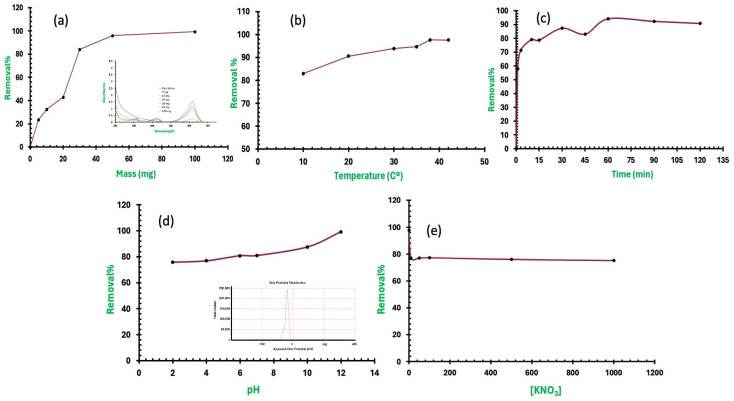
(**a**) Effect of Fe_3_O_4_/Ti_3_C_2_ nanocomposite dose on the MG removal; inset of the UV–Vis. spectra of the removal process of MG dye by different doses from Fe_3_O_4_/Ti_3_C_2_ nanocomposite; (**b**) effect of solution temperature on the removal of MG by Fe_3_O_4_/Ti_3_C_2_ nanocomposite; (**c**) impact of interaction time on MG elimination by Fe_3_O_4_/Ti_3_C_2_ nanocomposite removal efficiency; (**d**) influence of pH of the solution on the Fe_3_O_4_/Ti_3_C_2_ nanocomposite’s efficiency to remove MG, and inset of the Zeta potential value of Fe_3_O_4_/Ti_3_C_2_ nanocomposite; and (**e**) impact of solution ionic strength on removal of MG dye using Fe_3_O_4_/Ti_3_C_2_ nanocomposite.

The effects of several operating factors were investigated to ascertain the efficacy of Fe_3_O_4_/Ti_3_C_2_ nanocomposite solid adsorbent concerning the MG dye’s adsorption under diverse conditions, and they are detailed in the following parts. We investigated the amount of absorption of prepared nanomaterials for MG dye at a concentration of 10 mg/l in deionized water. The highest removal ratio (47.72%) of this dye was achieved by the Fe_3_O_4_/Ti_3_C_2_ nanocomposite. Figure 5a shows how the dosage results for the Fe_3_O_4_/Ti_3_C_2_ nanocomposite affect the removal efficiency and its UV–Vis. absorbance spectra (inset). Using 100 mg of Fe_3_O_4_/Ti_3_C_2_ nanocomposite, the maximum removal capacity was found to be 0.993 mg/g of MG, with a removal efficacy of 99.28%. While utilizing 5 mg of Fe_3_O_4_/Ti_3_C_2_ nanocomposite, the optimum removal capacity was found to be 4.68 mg/g, with a removal efficacy of 23.38%. MG clearance using Fe_3_O_4_/Ti_3_C_2_ nanocomposite increased with increasing Fe_3_O_4_/Ti_3_C_2_ nanocomposite dose, possibly due to the improved accessibility to extra adsorption active sites as a result of the increased adsorbent dose. Furthermore, 30 mg of Fe_3_O_4_/Ti_3_C_2_ nanocomposite was used for further experiments.

Temperature’s effect on the adsorption of MG utilizing Fe_3_O_4_/Ti_3_C_2_ nanocomposite was examined, and Figure 5b displays the findings. The figure makes it evident that the endothermic nature of the adsorption process is present because the percentage of MG adsorbed on the Fe_3_O_4_/Ti_3_C_2_ nanocomposite increases as temperature rises, which will be evaluated thoroughly regarding the thermodynamic parts. This is explained by the fact that more MG molecules were able to obtain enough energy to engage with the Fe_3_O_4_/Ti_3_C_2_ nanocomposite surface, which increased MG molecule mobility and diffusion. Furthermore, because of active diffusion, a temperature increase would cause pore sizes to grow, adding additional surface area for adsorption [33]. 

The duration of interaction between the solid adsorbents, Fe_3_O_4_/Ti_3_C_2_ nanocomposite, the adsorbate, and MG dye molecules is one of the critical operating parameters influencing the removal process. The results of this investigation on the impact of interaction time are shown in Figure 5c. The Fe_3_O_4_/Ti_3_C_2_ nanocomposite showed extremely rapid adsorption; full saturation happened in less than 1 h, and it had a removal capacity (q_t_) of (3.14 mg MG each g of Fe_3_O_4_/Ti_3_C_2_ nanocomposite) because of the increase in active sites associated with increasing the mass of the Fe_3_O_4_/Ti_3_C_2_ nanocomposite and consequent electrostatic interaction between the negative Fe_3_O_4_/Ti_3_C_2_ nanocomposite surface and the MG dye increased. Therefore, the amount of MG adsorbed on the Fe_3_O_4_/Ti_3_C_2_ nanocomposite rises with increasing contact time. An increase in interaction duration is correlated with a small increase in removal efficiency and capacity. Furthermore, a 60 min duration of the interaction between MG dye and Fe_3_O_4_/Ti_3_C_2_ nanocomposite was used for further experiments. 

Any adsorption/removal process that involves controlling surface charges of adsorbents, which changed according to pH value, depends heavily on the solution pH value. Surface charge is affected principally by the solution pH, which modifies the electrostatic conditions between adsorbent and adsorbate. The current results show that the adsorption of MG dye molecules onto Fe_3_O_4_/Ti_3_C_2_ nanocomposite increases when pH increases, indicating the degree of the surface charge that is negative. The information is consistent with the theory that adsorption is mostly caused by electrostatic attraction. The Fe_3_O_4_/Ti_3_C_2_ nanocomposite exhibited a noteworthy improvement in MG dye removal efficiency (87.63–99.7%) in Figure 5d when the pH of the solution was raised gradually from 2.0 to 12.0. This could be explained by electrostatic attraction between the MG neutral and cationic dye molecules (p*K*_a_ value 10.3) and the negatively charged Fe_3_O_4_/Ti_3_C_2_ nanocomposite surface (zeta potential value of −21 mV). In the meantime, the Fe_3_O_4_/Ti_3_C_2_ nanocomposite dramatically increased its ability to remove MG dye at a pH of 6.0, 80.88%, which in turn increased more through raising the pH to 12.0 (99.28%). The electrostatic attraction between the positively charged MG molecules and the negatively charged Fe_3_O_4_/Ti_3_C_2_ nanocomposite surface may be the cause of this phenomenon. This result demonstrates that the Fe_3_O_4_/Ti_3_C_2_ nanocomposite solid adsorbent’s mechanism for removing the MG molecules is electrostatic in nature. The existence of this electrostatic mechanism was demonstrated and confirmed by investigating the Fe_3_O_4_/Ti_3_C_2_ nanocomposite’s electrostatic interaction mechanism for the adsorption and removal of MG dye through varying the KNO_3_ concentration in the investigated solution and performing the removal experiment at different ionic strength values. Figure 5e illustrates how, when the (KNO_3_) concentration in the solution was increased to 5.0 mM, the percentage of MG dye removed dropped sharply to 76.89%, and further addition of KNO_3_ slightly retarded the adsorption mechanism; 0.1 M KNO_3_ was associated with 75.09% removal. The reason for this could be that the Fe_3_O_4_/Ti_3_C_2_ nanocomposite surface is surrounded by a double layer of positive ions (K^+^) and negative ions (NO_3_^−^) of (KNO_3_), which creates electrostatic repulsion with the MG dye molecules. This discovery supports the mechanism of electrostatic contact for the MG dye that had been removed by Fe_3_O_4_/Ti_3_C_2_ nanocomposite, which strongly corroborated earlier research that established the organic dyes’ electrostatic mechanism through solid clay-based adsorbents [34].

### 2.3. Kinetics Study

Information regarding the kinetics interaction between the adsorbent and adsorbate in the solid/solution system provides a comprehensive understanding of the possible interaction mechanism of adsorption in addition to the reaction pathways [35]. Several kinetics models assuming that the kinetic constants are time-invariant (a time-dependent system function that is not a direct function of time), such as pseudo-first-order (PFO) and pseudo-second-order (PSO), have been used for adsorption kinetics prediction at solid/solution interfaces. This assumption has been unacceptable when reactants (adsorbent and adsorbate) were spatially constrained by walls or phase boundaries [36]. Based on experimental and theoretical examinations, it was presented that the reaction rate coefficient is time-dependent (fractal-like kinetics), leading to the fractal-like pseudo-first-order (FL-PFO) and fractal-like pseudo-second-order (FL-PSO) kinetics models.

Therefore, the kinetic adsorption data were evaluated by using four nonlinear models: pseudo-first-order (PFO) [37], pseudo-second-order (PSO) [37], fractal-like pseudo-first-order (FL-PFO) [38], and fractal-like pseudo-second-order (FL-PSO) [38]. The mathematical equations of these respective models are shown in Equations (1)–(4).

(1) $$q_{t}=q_{e}\times [1-e^{-k_{1}t}]$$

(2) $$q_{t}=\frac{k_{2}{\times q}_{e}^{2}\times t}{1+q_{e}k_{2}\times t}$$

(3) $$q_{t}=q_{e}\times [1-e^{{\left(-k_{1,0}\times t\right)}^{n}}]$$

(4) $$q_{t}=\frac{k_{2,0}{\times q}_{e}^{2}\times t^{n}}{1+q_{e}k_{2,0}\times t^{n}}$$

where *t* is the interaction time (min); *q_t_*, and *q_e_* are the amount of MG dye adsorbed at time *t* and at equilibrium, respectively (mg g^−1^); *k*_1_ is the pseudo-first-order rate constant (min^−1^); *k*_2_ is the pseudo-second-order rate constant (g mg^−1^ min^−1^); *k*_1,0_ is the fractal-like pseudo-first-order constant rate (min^−1^), *k*_2,0_ is the fractal-like pseudo-second-order rate constant (g mg^−1^ min^−1^), and *n* is the fractional-like exponent (*n* > 0). The kinetic and equilibrium models’ appropriateness was then examined and evaluated using different statistical error functions: the residual sum of squares (*RSS*), Equation (5); the determination coefficient (*R*^2^), Equation (6); the adjusted determination coefficient (*R*^2^*_adj_*), Equation (7); the standard deviation of residues (*SD*), Equation (8); and the Bayesian Information Criterion (*BIC*), Equation (9) [37,39].

(5) $$RSS={\sum_{i}^{n}(q_{i,exp}-q_{i,model})}^{2}$$

(6) $$R^{2}=(\frac{{\sum_{i}^{n}(q_{i,exp}-{\bar{q}}_{i,exp})}^{2}-{\sum_{i}^{n}(q_{i,exp}-{\bar{q}}_{i,emodel})}^{2}}{{\sum_{i}^{n}(q_{i,exp}-{\bar{q}}_{i,exp})}^{2}})$$

(7) $$R_{adj}^{2}=1-(1-R^{2})(\frac{n-1}{n-p-1})$$

(8) $$SD=\sqrt{(\frac{1}{n-p}){\sum_{i}^{n}(q_{i,exp}-{\bar{q}}_{i,emodel})}^{2}}$$

(9) $$BIC=nLn\left(\frac{RSS}{n}\right)+pLn(n)$$

**Figure 6:**
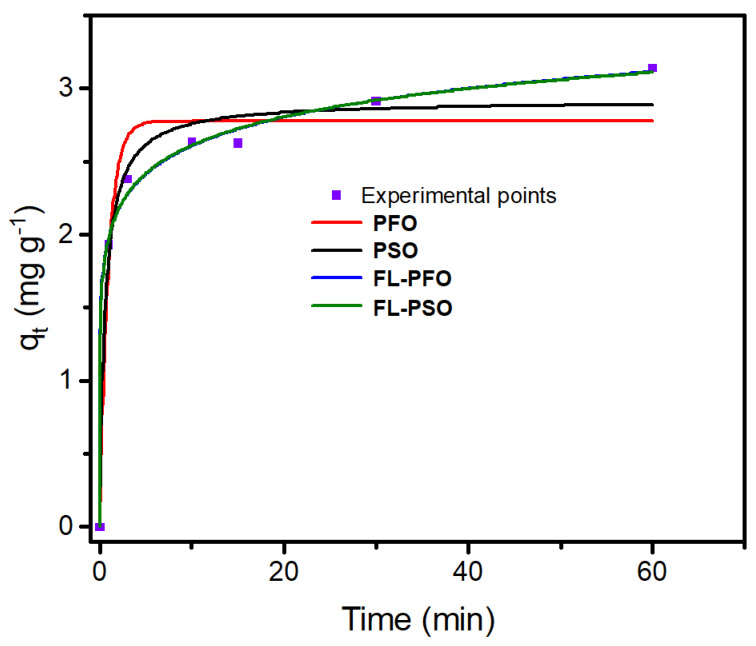
Kinetic curves for the adsorption removal of MG dye by Fe_3_O_4_/Ti_3_C_2_ nanocomposite adsorbent (conditions for the experiment: 10 mL, pH of 6.5, 30.0 mg Fe_3_O_4_/Ti_3_C_2_ nanocomposite, and 10.0 mg/L of MG dye).

In the above equations, *q*_*i*,*model*_ is the individual theoretical *q* value predicted by the model; *q*_*i*,*exp*_ is individual experimental *q* value; ${\bar{q}}_{i,exp}$ is the average of all experimental *q* values measured; *n* is the number of experiments; and *p* is the number of parameters in the fitting model. In this work, the $R_{adj}^{2}$, *SD*, and *BIC* values will be used to examine the appropriateness of different kinetics models, taking into consideration that the best-fitted model would have $R_{adj}^{2}$ value closer to unity, in addition to lower values of *SD* and *BIC*. Figure 6 shows the fitting of the four kinetics models with the experimental adsorption capacities (*q_t_*, mg/g) variation with interaction time (min), and Table 1 shows the kinetic parameters for each model. The four kinetics models were evaluated statistically based on the $R_{adj}^{2}$, *SD*, and *BIC* values to evaluate the most suitable model. Surprisingly, both the FL-PFO and FL-PSO kinetics models showed the best $R_{adj}^{2}$ values of 0.9944 and 0.9945, and the lowest standard deviation (*SD*) of residue values of 0.0790 and 0.0784, and the lowest *BIC* values of −31.67 and −31.78 for MG dye using Fe_3_O_4_/Ti_3_C_2_ nanocomposite as adsorbent (Table 1), respectively.

Furthermore, the process of adsorption/removal process is controlled/governed by diffusion kinetics too as the adsorption process occurs typically in four separate stages: mass action, bulk diffusion, liquid film diffusion, and intra-particle diffusion, which is sometimes referred to as pore and surface diffusion. The identification of the adsorption/diffusion process’s rate-determining step is the main focus of this research segment; based on the liquid film diffusion model, it predicts the rate processes’ kinetics by assuming that the adsorbate molecules’ slowest-moving stage in the adsorption process is their flow through a liquid film that surrounds the solid adsorbent, and intra-particle diffusion model assumes that the adsorbate molecules’ slowest-moving stage in the adsorption process is their flow through the solid adsorbent pores. 

**Figure 7:**
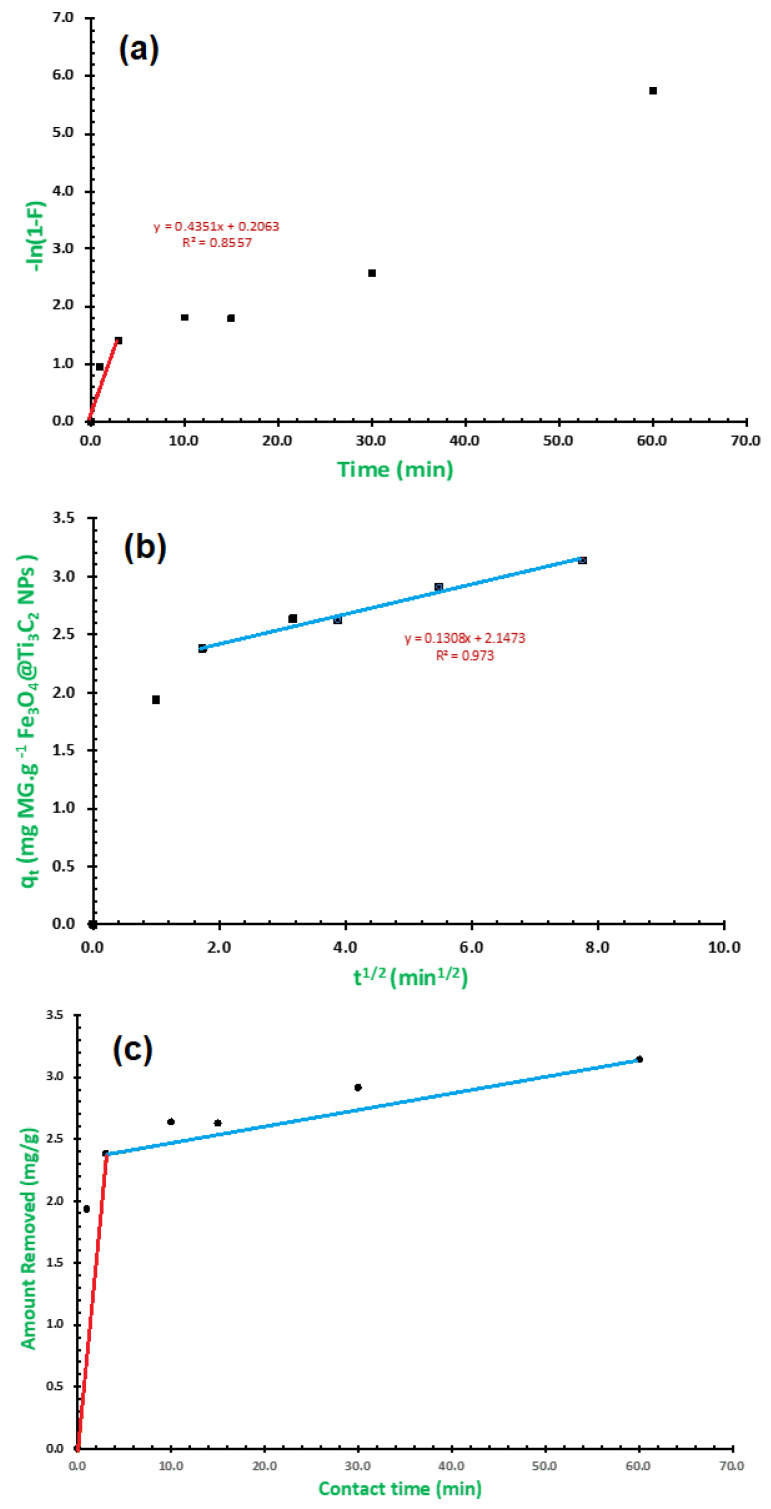
(**a**) Liquid film diffusion kinetics model, (**b**) intra-particle diffusion kinetics model, and (**c**) change over time in the quantity of MG dye adsorbed per unit mass of the Fe_3_O_4_/Ti_3_C_2_ nanocomposite.

The following equation [40] describes the liquid film diffusion model:

(10) $$Ln\left(1-F\right)=k_{fd}*t$$

where the film diffusion rate coefficient is denoted by *k_fd_* (min^−1^), and F denotes the fractional attainment of equilibrium (*F* = *q_t_*/*q_e_*). Figure 7a demonstrates how the liquid film diffusion model plot of [−*Ln* (1 − *F*)] against t converged when applied to the MG dye adsorption by Fe_3_O_4_/Ti_3_C_2_ nanocomposite, and a correlation coefficient of 0.856 was obtained, resulting in a straight line through the origin for the first 3 min. This suggests that the rate-determining step could be for the liquid film diffusion model within the first 3 min.

The following is the equation of the intra-particle diffusion model that Weber and Morris presented in 1963 [41]:

(11) $$q_{t}=k_{id}t^{1/2}+C$$

*C* is a constant inversely proportional to the boundary layer thickness (mg/g), and *k_id_* is the intra-particle diffusion rate constant (mg/g·min^1/2^). Upon utilizing the intra-particle diffusion model on the experimental data, the adsorption of the MG dye by the Fe_3_O_4_/Ti_3_C_2_ nanocomposite was plotted as *q_t_* versus *t*; the plot did not converge satisfactorily considering all the points, and, therefore, it was possible to draw a straight line through the origin, as observed in Figure 7b, obtained following the 3 initial minutes, with a correlation coefficient value of 0.973, indicating that the process that influences the rate may be the intra-particle diffusion model. Accordingly, both liquid film and intra-particle diffusion may have an impact on the removal diffusion kinetics in this work at the same time, as presented in Figure 7c.

### 2.4. Environmental Applications

**Figure 8:**
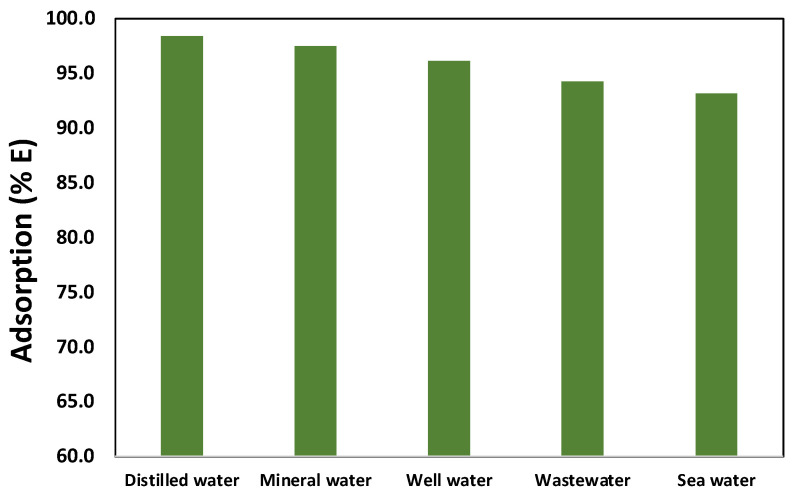
Fe_3_O_4_/Ti_3_C_2_ nanocomposite used to remove MG from water in the environment (conditions for the experiment: 10 mL solution, pH 8, 120 min, 50 mg Fe_3_O_4_/Ti_3_C_2_ nanocomposite, and 10 mg/L of MG dye).

Five separate real water samples were utilized to further confirm the effectiveness of the Fe_3_O_4_/Ti_3_C_2_ nanocomposite as an adsorbent for the removal of MG dye, and Figure 8 shows the results. After the real water sample collection, analysis was conducted on the MG dye concentrations, and it was discovered that they were below the detection limit; they were regarded as zero. Therefore, to simulate pollution and explore the possibility of using Fe_3_O_4_/Ti_3_C_2_ nanocomposite as a solid material for water pollution treatment, concentrated MG dye was added to real water samples until a final concentration of 10.0 mg/L was obtained. Afterward, the spiked real water samples were mixed with the Fe_3_O_4_/Ti_3_C_2_ nanocomposite. Regarding the MG dye, the samples of distilled water, mineral water, well water, wastewater, and seawater had removal percentages of 98.40%, 97.49%, 96.13%, 94.27%, and 93.14%, in that order. It is noteworthy that the minimal percentage of MG dye removal observed in the well water, wastewater, and seawater through the Fe_3_O_4_/Ti_3_C_2_ solid nanocomposite was mainly caused by the elevated concentrations of Na^+^, K^+^, Mg^2+^, and Ca^2+^ found in the selected real water samples as compared to the other real water samples. Because of these ions’ screening and shielding properties at such high concentrations, the amount of MG dye removed by the solid Fe_3_O_4_/Ti_3_C_2_ nanocomposite was significantly reduced. According to these findings, the Fe_3_O_4_/Ti_3_C_2_ nanocomposite was able to remove the majority of the MG dye from real environmental water samples.

### 2.5. Reusability Studies

For practical application, a solid adsorbent’s reusability is essential. From environmental and economic points of view, it is a highly crucial component of adsorption. Thus, the Fe_3_O_4_/Ti_3_C_2_ nanocomposite’s recycling effectiveness for MG dye adsorption was examined during four consecutive cycles. After each adsorption cycle, the MG dye molecules were adsorbed from the Fe_3_O_4/_Ti_3_C_2_ nanocomposite using a concentrated solution of NaCl; after that, the Fe_3_O_4/_Ti_3_C_2_ adsorbent was washed with acetone several times, and in the last step it was washed with deionized water and a centrifuge was used to separate the Fe_3_O_4/_Ti_3_C_2_ nanocomposite and then dried at room temperature before being used in another absorption cycle.

**Figure 9:**
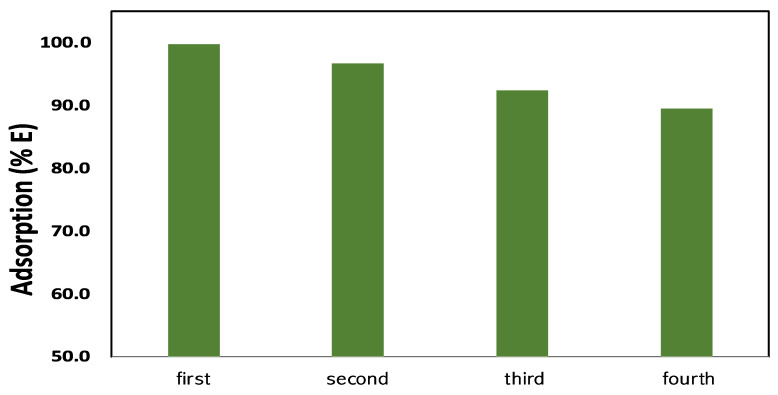
Reusability of the adsorbent Fe_3_O_4_/Ti_3_C_2_ nanocomposite in the extraction of MG dye from the solution (conditions for the experiment: 10 mL, pH 8, 120 min, 50 mg Fe_3_O_4_/Ti_3_C_2_ nanocomposite, and 10 mg/L of MG dye).

We discovered that, even after the four cycles with a high removal efficiency, the proportion of MG that was removed using the Fe_3_O_4_/Ti_3_C_2_ nanocomposite had somewhat decreased (Figure 9). It is clear from this that the Fe_3_O_4_/Ti_3_C_2_ nanocomposite is a reusable adsorbent for environmental cleanup.

### 2.6. Studies on Thermodynamics of the Removal

The three essential thermodynamic functions are entropy change (ΔS), enthalpy change (ΔH), and Gibbs free energy change (ΔG), which must be explored and measured to estimate thermodynamic viability and spontaneity using any physical or chemical procedure, such as using a solid adsorbent Fe_3_O_4_/Ti_3_C_2_ nanocomposite to remove MG dye from an aqueous solution. To determine the thermodynamic parameters, the following formulas were employed [42]:

(12) $$K_{d}=\frac{q_{t}}{C_{t}}\times 1000$$

(13) $${lnK}_{d}=\frac{\Delta S}{R}-\frac{\Delta H}{RT}$$

**Figure 10:**
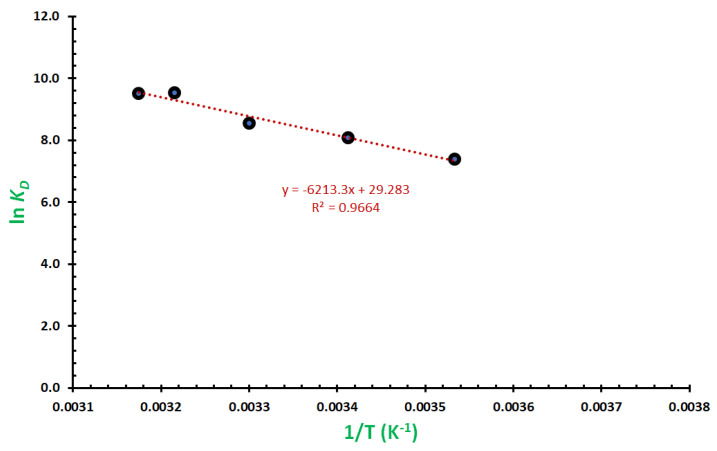
The calculations of thermodynamic parameters for the adsorption of MG dye from a model solution of Fe_3_O_4_/Ti_3_C_2_ nanocomposite.

ΔG = ΔH – TΔS(14)

where K_d_, the dimensionless thermodynamic distribution coefficient, is calculated at equilibrium concerning the amount of MG dye removed (mg of MG dye for each gram of Fe_3_O_4_/Ti_3_C_2_ nanocomposite), and the equilibrium concentration of MG dye in the solution is represented by C_t_ (mg/L). Figure 10 shows that the plot of ln K_d_ vs. 1/T revealed a straight line, and the ΔH and ΔS were calculated using the straight-line slope and intercept, in that order. Fe_3_O_4_/Ti_3_C_2_ nanocomposite’s thermodynamic characteristics for MG dye elimination were calculated, where the corresponding values for ∆H, ∆S, and ∆G were +49.07 kJ/mole, +234.29 J/mole.K, and −20.75 kJ/mole (at 298 K). There was a positive value obtained for the entropy change, demonstrating an increase in the degrees of freedom at the boundary between liquid and solid due to the MG dye attaching itself to the Fe_3_O_4_/Ti_3_C_2_ nanocomposite surface, promoting the removal process’ spontaneity, whereas the enthalpy change had a positive value, indicating the endothermic nature of the removal process, which unfavored the removal process. As a result, a negative change in the free energy would be anticipated for a process of spontaneous elimination. Conversely, the removal of MG dye from aqueous solution by Fe_3_O_4_/Ti_3_C_2_ nanocomposite is an entropy-driven process, as indicated by the positive values of ∆H, ∆S, and the negative value of ∆G [43].

Based on the above results, the removal/adsorption mechanism of the MG dye from aqueous solution by Fe_3_O_4_/Ti_3_C_2_ nanocomposite was found to be electrostatic in nature due to the attraction forces arising between the MG neutral and cationic dye molecules to the negatively charged Fe_3_O_4_/Ti_3_C_2_ nanocomposite surface, which were confirmed by studying the effect of the solution pH and ionic strength. Also, the removal process was well-described by both the FL-PFO and FL-PSO kinetics models and controlled by both liquid film and intra-particle diffusion kinetics. In addition, the removal process was entropy-driven, as indicated by the positive values of ∆H, ∆S, and the negative value of ∆G.

Based on the above-mentioned results, the maximum removal capacity of MG dye (10.0 mg/L), obtained using 0.5 g/L loading of Fe_3_O_4_/Ti_3_C_2_ nanocomposite (5 mg/10 mL) within 60.0 min, was found to be 4.68 mg/g, which is very competitive with other removal methods based on removal capacity and treatment time, as presented in Table 2.

## 3. Experimental

### 3.1. Substances

All studies were conducted using chemicals of analytical grade: MAX phase powder of Ti_3_AlC_2_ (NANOSHEL, Dera Bassi, India, purity: 99%); 48.051% hydrofluoric acid (HF, Parmcac Applichem IT Reagents, Darmstadt, Germany); polyethylene glycol (PEG 4000, BOC Sciences, New York, NY, USA); and 99.5% acetone (Abuljadayel & Sons Co., Jeddah, Saudi Arabia).

### 3.2. Fe_3_O_4_ Nanoparticles and MXene Nanosheet Synthesis

Powdered Ti_3_AlC_2_ (>98 weight percent pure) was offered commercially by Forsman Com., Ltd. (Shanghai, China) to produce Fe_3_O_4_ and Ti_3_C_2_ MXene nanoparticles. In order to remove the Al layers completely, 2 g of the Ti_3_AlC_2_ powder was stirred for 24 h at 60 °C while submerged in HF solutions (20 mL; >40 wt%). After removing the HF, the solution was centrifuged and repeatedly washed with deionized water until the pH reached 5–6. The generated Ti_3_C_2_ powdered form was dried under a vacuum at 80 °C for a whole day. The process of hydrothermal synthesis was used to create Fe_3_O_4_ nanoparticles. First, under vigorous stirring conditions for 30 min, 0.43 g of NaOH, 1.35 g of FeCl_3_.6H_2_O, and 10 g of polyethylene glycol (PEG 4000, BOC Sciences, New York, NY, USA) were dissolved in 40 mL of ethylene glycol. The solution was then instantly put into a 100 mL Teflon-lined autoclave and heated at 200 °C for 8 h. In order to get rid of the contaminant, the sediment was repeatedly cleaned with ethanol and distilled water. The Fe_3_O_4_ nanoparticles that were produced were then dried in a vacuum for 6 h at 80 °C.

### 3.3. Synthesis of Fe_3_O_4_/Ti_3_C_2_ Nanocomposite

For 30 min, deionized water was used to ultrasonically disperse 100 mg of Ti_3_C_2_ and 40 mg of Fe_3_O_4_ nanoparticles in 80 mL and 20 mL, respectively. Both were then combined, and they underwent 6 h ultra-sonification therapy after that. The Ti_3_C_2_/Fe_3_O_4_ nanocomposite was then obtained by filtering the suspension. The finished nanocomposite was dried in a vacuum for 24 h at 80 °C.

### 3.4. Characterizations

The crystalline structures of all the samples were analyzed using powder X-ray diffraction (XRD, Cu K radiation, Bruker D2 Phaser X-ray diffractometer) with a step scan of 0.02° per step. Scanning electron microscopy (SEM, Gemini, Zeiss-Ultra 55, Chiyoda, Tokyo) and transmission electron microscopy (TEM, Sirion 200FEI, Eindhoven, The Netherlands) were used to examine the microstructure and morphology of produced samples. The FT-IR spectra of the samples were recorded using an FT-IR spectrophotometer (Spectrum 100, Perkin Elmer, Waltham, MA, USA). NOVA3200e (Quantachrome, Boynton Beach, FL, USA) was utilized to determine the BET specific surface area of the materials by utilizing the nitrogen adsorption/desorption isotherm at 77 K.

### 3.5. Adsorption Experiment

First, the prepared nanomaterials studied the adsorption of 10 mL of 10 mg/L of malachite green dye for a certain period. After the experiment began, at a wavelength of 617 nm, the SHIMADZU CPS-240A UV–Vis (Kyoto, Japan) was employed to measure the quantity of adsorbed MG dye. The effects of experimental parameters such as Fe_3_O_4_/Ti_3_C_2_ nanocomposite mass, solution pH, initial MG dye concentration, solution temperature, and ionic strength were examined to estimate the efficacy of the nanocomposite adsorbent toward the adsorption of MG dye under various conditions in model and real water samples. The efficiency of the Fe_3_O_4_/Ti_3_C_2_ nanocomposite towards the removal/adsorption of MG dye was calculated using Equation (15), and the removal capacity (*q_t_,* mg/g) was estimated using Equation (16).

(15) $$\%\;adsorption=\frac{C_{o}-C_{t}}{C_{o}}$$

(16) $$q_{t}=\frac{V*\left(C_{o}-C_{t}\right)}{m}$$

where *V* is the volume of the solution (L), m is the dose (g) of the Fe_3_O_4_/Ti_3_C_2_ nanocomposite, and *C*_0_ and *C_t_* are the concentrations of the MG dye in the solution at time zero and any time thereafter.

### 3.6. Collecting Real Water Samples

Several real water samples were gathered to confirm the Fe_3_O_4_/Ti_3_C_2_ nanocomposite’s suitability as a solid adsorbent for the environmental cleanup of contaminated real water using MG dye. A wastewater sample was obtained from King Abdelaziz University (KAUWW), Jeddah City’s Membrane Bio-Reactor Technology Wastewater Treatment Plant (MBR 6000 STP) (Latitude deg. North 21.487954, Longitude deg. East 39.236748). At Abdraboh AL-Beladi Farm in the Khulais Province of Saudi Arabia, a deep well (latitude deg. North 22.124376, longitude deg. East 39.500618) was used to gather the well water sample. A sample of distilled water from a distillation unit from King Abdelaziz University’s laboratories also included a Row mineral water sample (commercial water in bottles) and a sample from the Red Sea. A 45.0 µm Millipore filter paper was used to filter all real water samples, which were then stored in Teflon^®^ bottles at 5 °C in the dark.

## 4. Conclusions 

This study explored the preparation of the modified magnetite/MXene nanocomposite and its application for the removal/adsorption of malachite green dye from model solutions and environmental real water samples. The prepared Fe_3_O_4_/Ti_3_C_2_ nanocomposite was physically and chemically characterized using XRD, SEM, TEM, FT-IR, and surface area analysis, and the results demonstrated the successful preparation of MXene and magnetite, in addition to the homogenous distribution of Fe_3_O_4_ over the MXene surface. The removal/adsorption was explored, and the results revealed maximum removal achieved using 100 mg of the Fe_3_O_4_/Ti_3_C_2_ nanocomposite within 60 min under ambient conditions, with an optimum removal capacity of 4.676 mg/g using 5 mg only. Also, removal was studied kinetically, and the results revealed that both the fractal-like pseudo-first-order and fractal-like pseudo-second-order kinetics models could better explain the removal of MG dye by the Fe_3_O_4_/Ti_3_C_2_ nanocomposite. Also, the Fe_3_O_4_/Ti_3_C_2_nanocomposite was used for the MG dye removal/adsorption from spiked real environmental water samples collected from different places, and the results demonstrated extraordinary efficacy of the Fe_3_O_4_/Ti_3_C_2_ nanocomposite and the prospective application of the Fe_3_O_4_/Ti_3_C_2_ nanocomposite for the environmental remediation of polluted water. The recycling and reuse of the Fe_3_O_4_/Ti_3_C_2_ nanocomposite for the successive removal of the MG dye was studied, and the results showed the possibility of reusing the Fe_3_O_4_/Ti_3_C_2_ nanocomposite for four consecutive times with high efficiency. The thermodynamics of the removal/adsorption process were also explored and the results demonstrated the spontaneity of the process: a negative Gibbs free energy change value of −20.75 kJ/mole, a positive entropy change value of +234.29 J/mole.K, and a positive enthalpy change value of +49.07 kJ/mole, indicating that the removal/adsorption of the MG dye by the Fe_3_O_4_/Ti_3_C_2_nanocomposite is an entropy-driven process.

## Figures and Tables

**Table 1:** Parameters of the kinetics models for the removal of MG dye from model solutions using Fe_3_O_4_/Ti_3_C_2_ nanocomposite.

**Pseudo-First-Order Kinetics Model (PFO)**							
***q*_*e*,*exp*_ (mg g^−1^)**	***q*_*e*,*cal*_ (mg g^−1^)**	***k*_1_ (min^−1^)**	***R* ^2^**	$R_{adj}^{2}$	**SD**	**RSS**	**BIC**
3.140	2.776	1.080	0.9573	0.9488	0.2392	0.2861	−16.544
**Pseudo-second-order kinetics model (PSO)**							
***q*_*e*,*exp*_ (mg g^−1^)**	***q*_*e*,*cal*_ (mg g^−1^)**	***k*_2_ (g mg^−1^ min^−1^)**	***R* ^2^**	$R_{adj}^{2}$	**SD**	**RSS**	**BIC**
3.140	2.917	0.5965	0.9813	0.9775	0.1584	0.1254	−22.315
**Fractal-like Pseudo-first-order Kinetics model (FL-PFO)**							
***q*_*e*,*exp*_ (mg g^−1^)**	***q*_*e*,*cal*_ (mg g^−1^)**	***k*_1,0_ (min^−1^)**	***R* ^2^**	$R_{adj}^{2}$	**SD**	**RSS**	**BIC**
3.140	4.446	0.0488	0.9963	0.9944	0.0790	0.0249	−31.668
**Fractal-like Pseudo-second-order Kinetics model (FL-PSO)**							
***q*_*e*,*exp*_ (mg g^−1^)**	***q*_*e*,*cal*_ (mg g^−1^)**	***k*_2,0_ (g mg^−1^ min^−1^)**	***R* ^2^**	$R_{adj}^{2}$	**SD**	**RSS**	**BIC**
3.140	5.358	0.1098	0.9963	0.9945	0.0784	0.0246	−31.783

**Table 2:** Removal capacity of MG dye by various removal techniques.

Material	Removal Capacity (mg/g)	Removal Time	Reference
Bottom ash	0.71	83 min	[44]
Polyethylene glycol micelles	1.00	10 min	[45]
Unsaturated Polyester Ce(IV) phosphate	1.01	35 min	[46]
Fum rosin alcoholpoly(acrylamide)	1.40	300 min	[47]
Hybrid nanocomposite	3.21	28 h	[48]
Neem sawdust	4.35	14 min	[49]
Fe_3_O_4_/Ti_3_C_2_ nanocomposite	4.68	60 min	[Current study]
TiO_2_ nanoparticles	6.25	40 min	[50]
Deinococcus radiodurans	7.63	30 min	[51]

## Data Availability

The data presented in this study are available in article.

## References

[1] Al-Tohamy R., Ali S.S., Li F., Okasha K.M., Mahmoud Y.A.-G., Elsamahy T., Jiao H., Fu Y., Sun J. (2022). A critical review on the treatment of dye-containing wastewater: Ecotoxicological and health concerns of textile dyes and possible remediation approaches for environmental safety. Ecotoxicol. Environ. Saf..

[2] Jiang F., Dinh D.M., Hsieh Y.-L. (2017). Adsorption, and desorption of cationic malachite green dye on cellulose nanofibril aerogels. Carbohydr. Polym..

[3] Chouli F., Ezzat A.O., Sabantina L., Benyoucef A., Zehhaf A. (2024). Optimization Conditions of Malachite Green Adsorption onto Almond Shell Carbon Waste Using Process Design. Molecules.

[4] Nayl A.A., Abd-Elhamid A.I., Arafa W.A.A., Ahmed I.M., El-Shanshory A.A., Abu-Saied M.A., Soliman H.M.A., Abdelgawad M.A., Ali H.M., Bräse S. (2022). Chitosan-Functionalized-Graphene Oxide (GO@CS) Beads as an Effective Adsorbent to Remove Cationic Dye from Wastewater. Polymers.

[5] Zhang H., Zhang T., Ding S., Wang X. (2023). Development of loose thin film nanofibrous composite nanofiltration membrane with modified g-C3N4 nanosheets barrier layer for efficient separation of salt/dye mixtures. Sep. Purif. Technol..

[6] Akpomie K.G., Conradie J. (2020). Advances in application of cotton-based adsorbents for heavy metals trapping, surface modifications and future perspectives. Ecotoxicol. Environ. Saf..

[7] He H., Wang X., Yu Q., Wu W., Feng X., Kong D., Ren X., Gao J. (2023). In Situ Growth of Ti_3_C_2_/UiO-66-NH_2_ Composites for Photoreduction of Cr (VI). Catalysts.

[8] Yang G., Li S., Wang X., Ding B., Li Y., Lin H., Tang D., Ren X., Wang Q., Luo S. (2021). A universal strategy boosting photoelectrochemical water oxidation by utilizing MXene nanosheets as hole transfer mediators. Appl. Catal. B Environ..

[9] Naguib M., Kurtoglu M., Presser V., Lu J., Niu J., Heon M., Hultman L., Gogotsi Y., Barsoum M.W. (2011). Two-Dimensional Nanocrystals Produced by Exfoliation of Ti_3_AlC_2_. Adv. Mater..

[10] Wu Z., Shen J., Li C., Zhang C., Wu C., Li Z., An X., He L. (2023). Niche Applications of MXene Materials in Photothermal Catalysis. Chemistry.

[11] Wu Z., Shen J., Li C., Zhang C., Feng K., Wang Z., Wang X., Meira D.M., Cai M., Zhang D. (2023). Mo_2_TiC_2_ MXene-Supported Ru Clusters for Efficient Photothermal Reverse Water–Gas Shift. ACS Nano.

[12] Chen S.-y., Deng Y.-f., Huang T., Zhang N., Wang Y. (2024). Polydopamine-assisted MXene decoration on electrospun polylactide fibers toward oil/water separation and organic dye adsorption. Sep. Purif. Technol..

[13] Yang F., Li J., Dong J., Chen S., Hu W., Zhang Y., Wang H., Li Z., Wang Z. (2024). MX@MIL-125(Ti)-mediated sonocatalytic degradation for the dyes and microplastics. Sep. Purif. Technol..

[14] Tran N.M., Ta Q.T.H., Sreedhar A., Noh J.-S. (2021). Ti_3_C_2_T_x_ MXene playing as a strong methylene blue adsorbent in wastewater. Appl. Surf. Sci..

[15] Katheresan V., Kansedo J., Lau S.Y. (2018). Efficiency of various recent wastewater dye removal methods: A review. J. Environ. Chem. Eng..

[16] Zhang S., Liao S., Qi F., Liu R., Xiao T., Hu J., Li K., Wang R., Min Y. (2020). Direct deposition of two-dimensional MXene nanosheets on commercially available filter for fast and efficient dye removal. J. Hazard. Mater..

[17] Wu Z., Li C., Li Z., Feng K., Cai M., Zhang D., Wang S., Chu M., Zhang C., Shen J. (2021). Niobium and Titanium Carbides (MXenes) as Superior Photothermal Supports for CO_2_ Photocatalysis. ACS Nano.

[18] Tawalbeh M., Mohammed S., Al-Othman A., Yusuf M., Mofijur M., Kamyab H. (2023). MXenes and MXene-based materials for removal of pharmaceutical compounds from wastewater: Critical review. Environ. Res..

[19] Gopalram K., Kapoor A., Kumar P.S., Sunil A., Rangasamy G. (2023). MXenes and MXene-Based Materials for Removal and Detection of Water Contaminants: A Review. Ind. Eng. Chem. Res..

[20] Wang X., Xu Q., Zhang L., Pei L., Xue H., Li Z. (2023). Adsorption of methylene blue and Congo red from aqueous solution on 3D MXene/carbon foam hybrid aerogels: A study by experimental and statistical physics modeling. J. Environ. Chem. Eng..

[21] Elgarhy G.S., El-Subruiti G.M., Omer A.M., Eltaweil A.S. (2024). 2D/3D MXene/NiFeMn-layered double hydroxide decorated gelatin for removal of Cr (VI) and Congo red: Performance and mechanism. J. Mol. Liq..

[22] Xue H., Gao X., Seliem M.K., Mobarak M., Dong R., Wang X., Fu K., Li Q., Li Z. (2023). Efficient adsorption of anionic azo dyes on porous heterostructured MXene/biomass activated carbon composites: Experiments, characterization, and theoretical analysis via advanced statistical physics models. Chem. Eng. J..

[23] Liu D., Li T., Sun W., Zhou W., Zhang G. (2022). Magnetic Ti_3_C_2_ MXene Nanomaterials for Doxorubicin Adsorption from Aqueous Solutions: Kinetic, Isotherms, and Thermodynamic Studies. ACS Omega.

[24] Sharifi M.J., Nouralishahi A., Hallajisani A. (2023). Fe_3_O_4_-chitosan nanocomposite as a magnetic biosorbent for removal of nickel and cobalt heavy metals from polluted water. Int. J. Biol. Macromol..

[25] Elgamal A.M., El-Ghany N.A.A., Saad G.R. (2023). Synthesis and characterization of hydrogel-based magnetite nanocomposite adsorbents for the potential removal of Acid Orange 10 dye and Cr (VI) ions from aqueous solution. Int. J. Biol. Macromol..

[26] Zawrah M.F., El-Gammal M.I., Salem M., El-Sonbati M.A., Ahmed M. (2023). Recycling of Rice Husk for Preparation of Activated Carbon/Magnetite Nanocomposites for Removal of Methylene Blue from Wastewater. Int. J. Environ. Res..

[27] Wang Y., Li Y., Qiu Z., Wu X., Zhou P., Zhou T., Zhao J., Miao Z., Zhou J., Zhuo S. (2018). Fe_3_O_4_@Ti_3_C_2_ MXene hybrids with ultrahigh volumetric capacity as an anode material for lithium-ion batteries. J. Mater. Chem. A.

[28] Yu H., Wang Y., Jing Y., Ma J., Du C.-F., Yan Q. (2019). Surface Modified MXene-Based Nanocomposites for Electrochemical Energy Conversion and Storage. Small.

[29] Rakhi B., Ahmed B., Hedhili M.N., Anjum D.H., Alshareef H.N. (2015). Effect of Postetch Annealing Gas Composition on the Structural and Electrochemical Properties of Ti_2_CT_x_ MXene Electrodes for Supercapacitor Applications. Chem. Mater..

[30] Scheibe B., Kupka V., Peplińska B., Jarek M., Tadyszak K. (2019). The Influence of Oxygen Concentration during MAX Phases (Ti_3_AlC_2_) Preparation on the α-Al_2_O_3_ Microparticles Content and Specific Surface Area of Multilayered MXenes (Ti_3_C_2_T_x_). Materials.

[31] Mahmood M., Rasheed A., Ayman I., Rasheed T., Munir S., Ajmal S., Agboola P.O., Warsi M.F., Shahid M. (2021). Synthesis of ultrathin MnO_2_ nanowire-intercalated 2D-MXenes for high-performance hybrid supercapacitors. Energy Fuels.

[32] Kiran N.U., Deore A.B., More M.A., Late D.J., Rout C.S., Mane P., Chakraborty B., Besra L., Chatterjee S. (2022). Comparative Study of Cold Electron Emission from 2D Ti_3_C_2_T_X_ MXene Nanosheets with Respect to Its Precursor Ti_3_SiC_2_ MAX Phase. ACS Appl. Electron. Mater..

[33] Woo S.-Y., Lee H.-S., Kim J.-S., Kim K.-H., Ji H., Kim Y.-D. (2022). Applicability assessment of functional adsorption zeolite materials in adsorption desalination cum cooling systems driven by low-grade heat source. Chem. Eng. J..

[34] Zhang T., Wang W., Zhao Y., Bai H., Wen T., Kang S., Song G., Song S., Komarneni S. (2021). Removal of heavy metals and dyes by clay-based adsorbents: From natural clays to 1D and 2D nanocomposites. Chem. Eng. J..

[35] Gupta S.S., Bhattacharyya K.G. (2011). Kinetics of adsorption of metal ions on inorganic materials: A review. Adv. Colloid Interface Sci..

[36] Kopelman R. (1988). Fractal Reaction Kinetics. Science.

[37] Lima E.C., Dehghani M.H., Guleria A., Sher F., Karri R.R., Dotto G.L., Tran H.N., Dehghani M.H., Karri R., Lima E.C. (2021). Adsorption: Fundamental aspects and applications of adsorption for effluent treatment. Green Technologies for the Defluoridation of Water.

[38] Haerifar M., Azizian S. (2014). Fractal-like kinetics for adsorption on heterogeneous solid surfaces. J. Phys. Chem. C.

[39] Lima E.C., Hosseini-Bandegharaei A., Moreno-Piraján J.C., Anastopoulos I. (2019). A critical review of the estimation of the thermodynamic parameters on adsorption equilibria. Wrong use of equilibrium constant in the Van’t Hoof equation for calculation of thermodynamic parameters of adsorption. J. Mol. Liq..

[40] Yao C., Chen T. (2017). A film-diffusion-based adsorption kinetic equation and its application. Chem. Eng. Res. Des..

[41] An B. (2020). Cu (II) and As (V) Adsorption Kinetic Characteristic of the Multifunctional Amino Groups in Chitosan. Processes.

[42] Owija N.Y., Salam S.K.M.A. (2021). Removal of cadmium ions from aqueous solution by Zero valent iron nanoparticles: Equilibrium and thermodynamic studies. J. Mol. Liq..

[43] Li C., Kong D., Yao X., Ma X., Wei C., Wang H. (2022). Adsorption Characteristics and Molecular Simulation of Malachite Green onto Modified Distillers’ Grains. Water.

[44] Gupta V., Mittal A., Krishnan L., Gajbe V. (2004). Adsorption kinetics and column operations for the removal and recovery of malachite green from wastewater using bottom ash. Sep. Purif. Technol..

[45] Chen J., Mao J., Mo X., Hang J., Yang M. (2009). Study of adsorption behavior of malachite green on polyethylene glycol micelles in cloud point extraction procedure. Colloids Surfaces A Physicochem. Eng. Asp..

[46] Khan A.A., Ahmad R., Khan A., Mondal P.K. (2013). Preparation of unsaturated polyester Ce (IV) phosphate by plastic waste bottles and its application for removal of malachite green dye from water samples. Arab. J. Chem..

[47] Kaith B.S., Jindal R., Sharma R. (2015). Synthesis of a *Gum rosin* alcohol-poly(acrylamide) based adsorbent and its application in removal of malachite green dye from waste water. RSC Adv..

[48] Kaith B.S., Sukriti Sharma J., Kaur T., Sethi S., Shanker U., Jassal V. (2016). Microwave-assisted green synthesis of hybrid nanocomposite: Removal of malachite green from waste water. Iran. Polym. J..

[49] Khattri S., Singh M. (2009). Removal of malachite green from dye wastewater using neem sawdust by adsorption. J. Hazard. Mater..

[50] Abou-Gamra Z.M., Ahmed M.A. (2015). TiO_2_ nanoparticles for removal of malachite green dye from waste water. Adv. Chem. Eng. Sci..

[51] Lv G.-Y., Cheng J.-H., Chen X.-Y., Zhang Z.-F., Fan L.-F. (2013). Biological decolorization of malachite green by *Deinococcus radiodurans* R1. Bioresour. Technol..

